# Supplementary material for: Type III TGF-β Receptor Down-Regulation Promoted Tumor Progression via Complement Component C5a Induction in Hepatocellular Carcinoma
Source: Cancers (Basel). 2021 Mar 25;13(7):1503. doi: 10.3390/cancers13071503 (PMC8037431; doi:10.3390/cancers13071503)
Supplement: Supplementary file 1 [file cancers-13-01503-s001.zip › Supplementary Figures.pptx]

## Slide 1
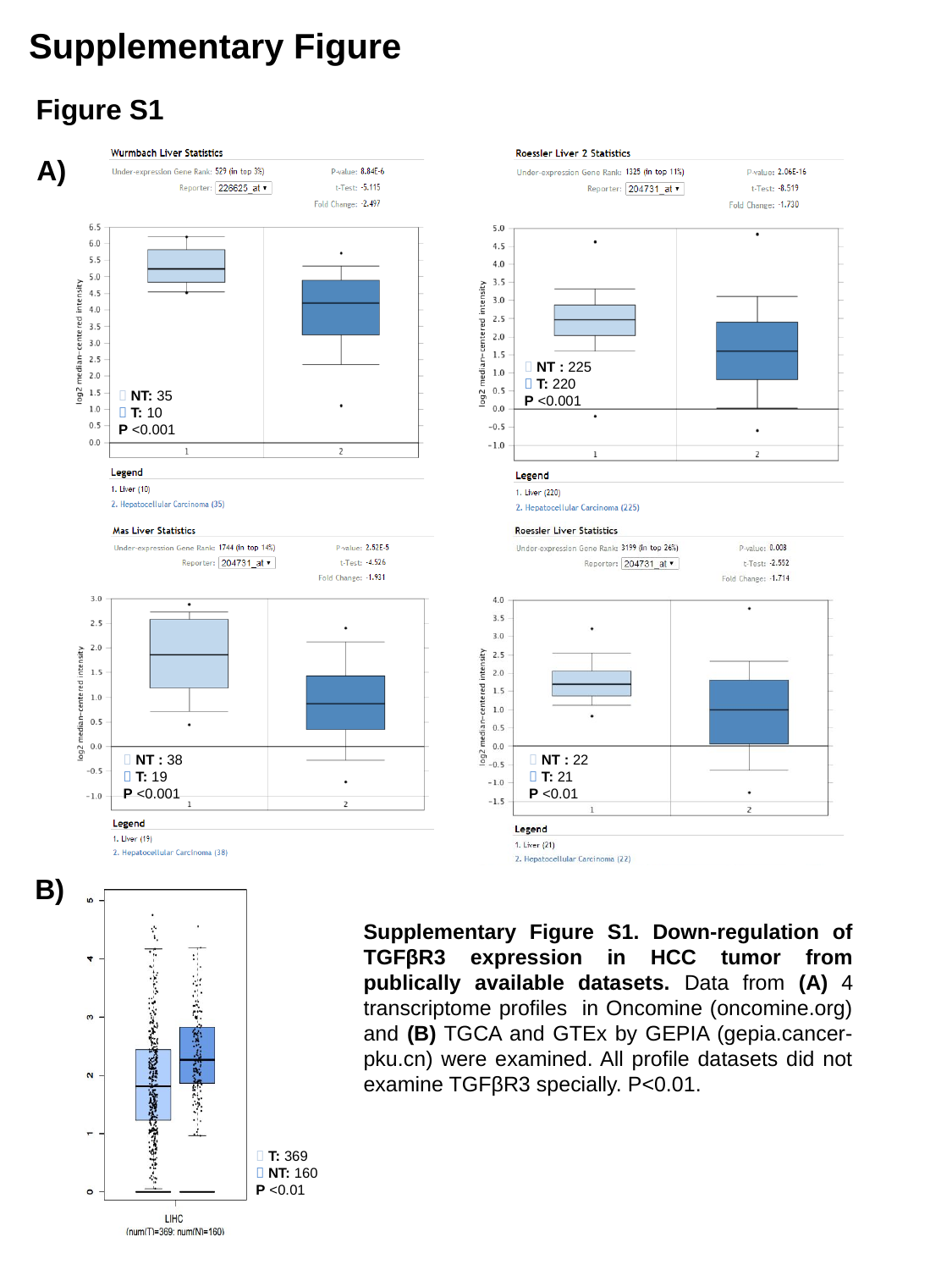

# Supplementary Figure
Figure S1
A)
 NT : 225
 T: 220
P <0.001
 NT: 35
 T: 10
P <0.001
 NT : 38
 T: 19
P <0.001
 NT : 22
 T: 21
P <0.01
B)
Supplementary Figure S1. Down-regulation of TGFβR3 expression in HCC tumor from publically available datasets. Data from (A) 4 transcriptome profiles in Oncomine (oncomine.org) and (B) TGCA and GTEx by GEPIA (gepia.cancer-pku.cn) were examined. All profile datasets did not examine TGFβR3 specially. P<0.01.
 T: 369
 NT: 160
P <0.01

## Slide 2
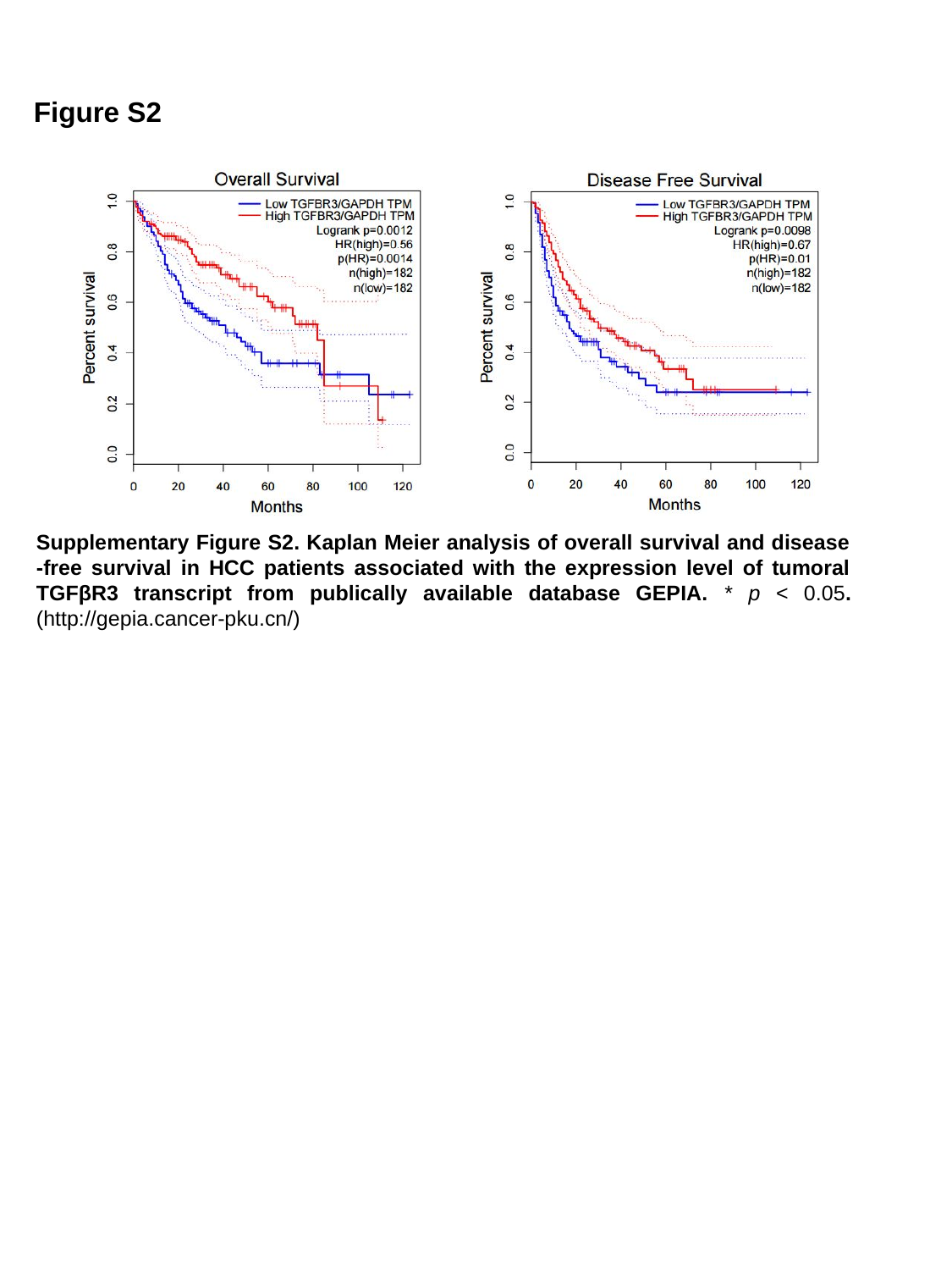

Figure S2
Supplementary Figure S2. Kaplan Meier analysis of overall survival and disease -free survival in HCC patients associated with the expression level of tumoral TGFβR3 transcript from publically available database GEPIA. * p < 0.05. (http://gepia.cancer-pku.cn/)

## Slide 3
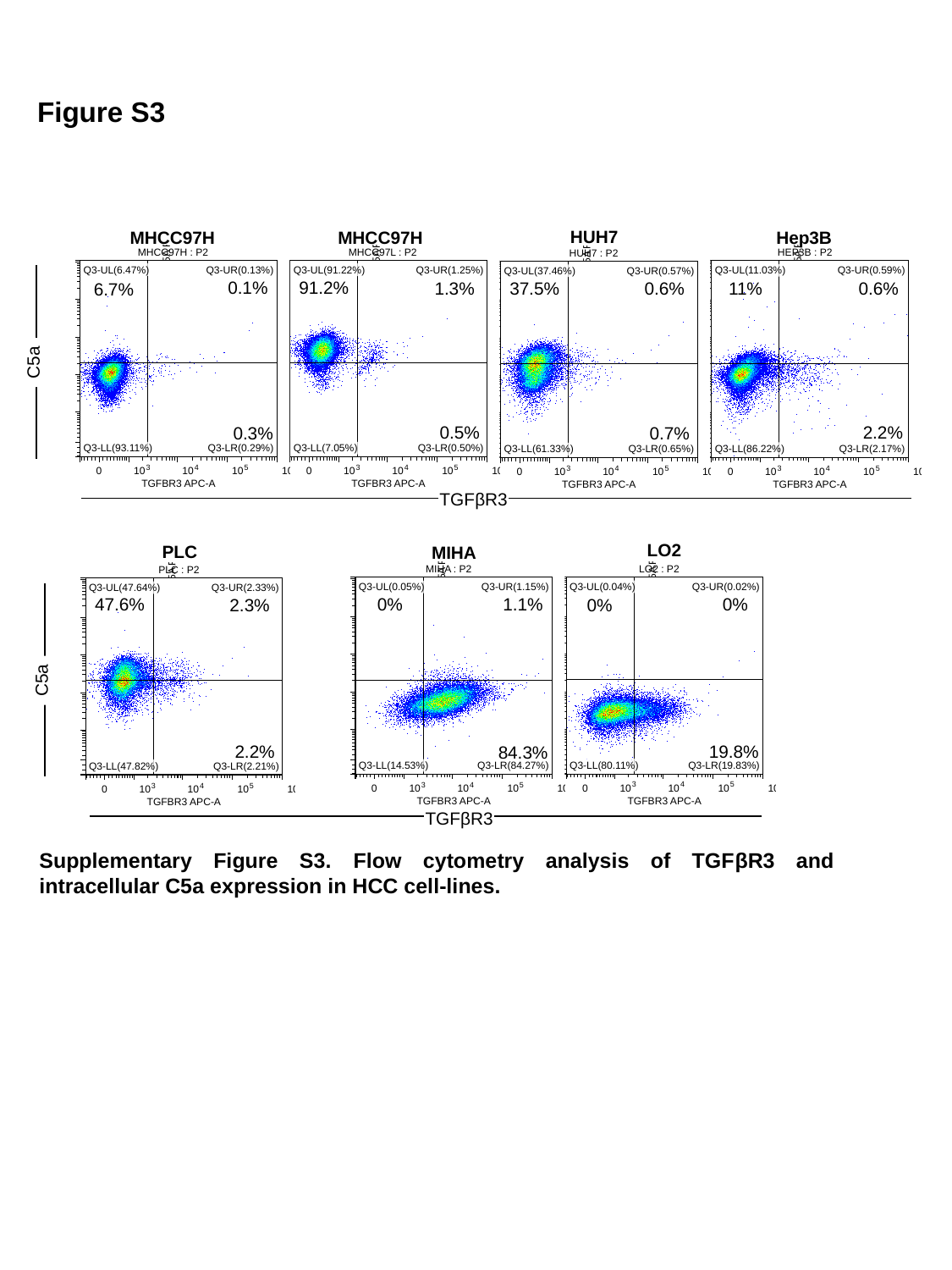

Figure S3
HUH7
Hep3B
MHCC97H
MHCC97H
91.2%
0.1%
37.5%
1.3%
0.6%
0.6%
11%
6.7%
C5a
0.5%
2.2%
0.7%
0.3%
TGFβR3
LO2
PLC
MIHA
47.6%
0%
0%
1.1%
0%
2.3%
C5a
19.8%
2.2%
84.3%
TGFβR3
Supplementary Figure S3. Flow cytometry analysis of TGFβR3 and intracellular C5a expression in HCC cell-lines.

## Slide 4
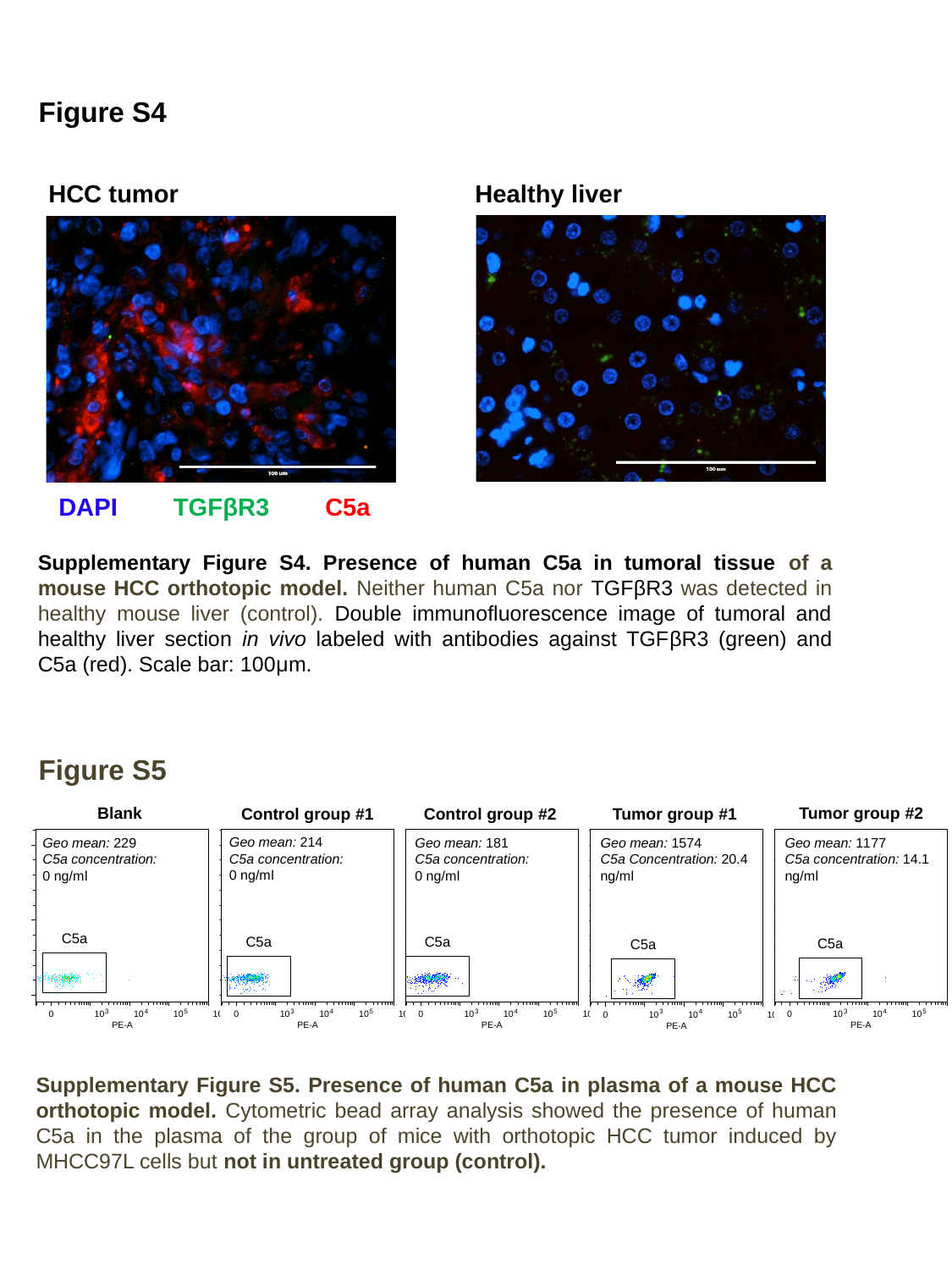

Figure S4
Healthy liver
HCC tumor
DAPI TGFβR3 C5a
Supplementary Figure S4. Presence of human C5a in tumoral tissue of a mouse HCC orthotopic model. Neither human C5a nor TGFβR3 was detected in healthy mouse liver (control). Double immunofluorescence image of tumoral and healthy liver section in vivo labeled with antibodies against TGFβR3 (green) and C5a (red). Scale bar: 100μm.
Figure S5
C5a
C5a
C5a
C5a
C5a
Supplementary Figure S5. Presence of human C5a in plasma of a mouse HCC orthotopic model. Cytometric bead array analysis showed the presence of human C5a in the plasma of the group of mice with orthotopic HCC tumor induced by MHCC97L cells but not in untreated group (control).

## Slide 5
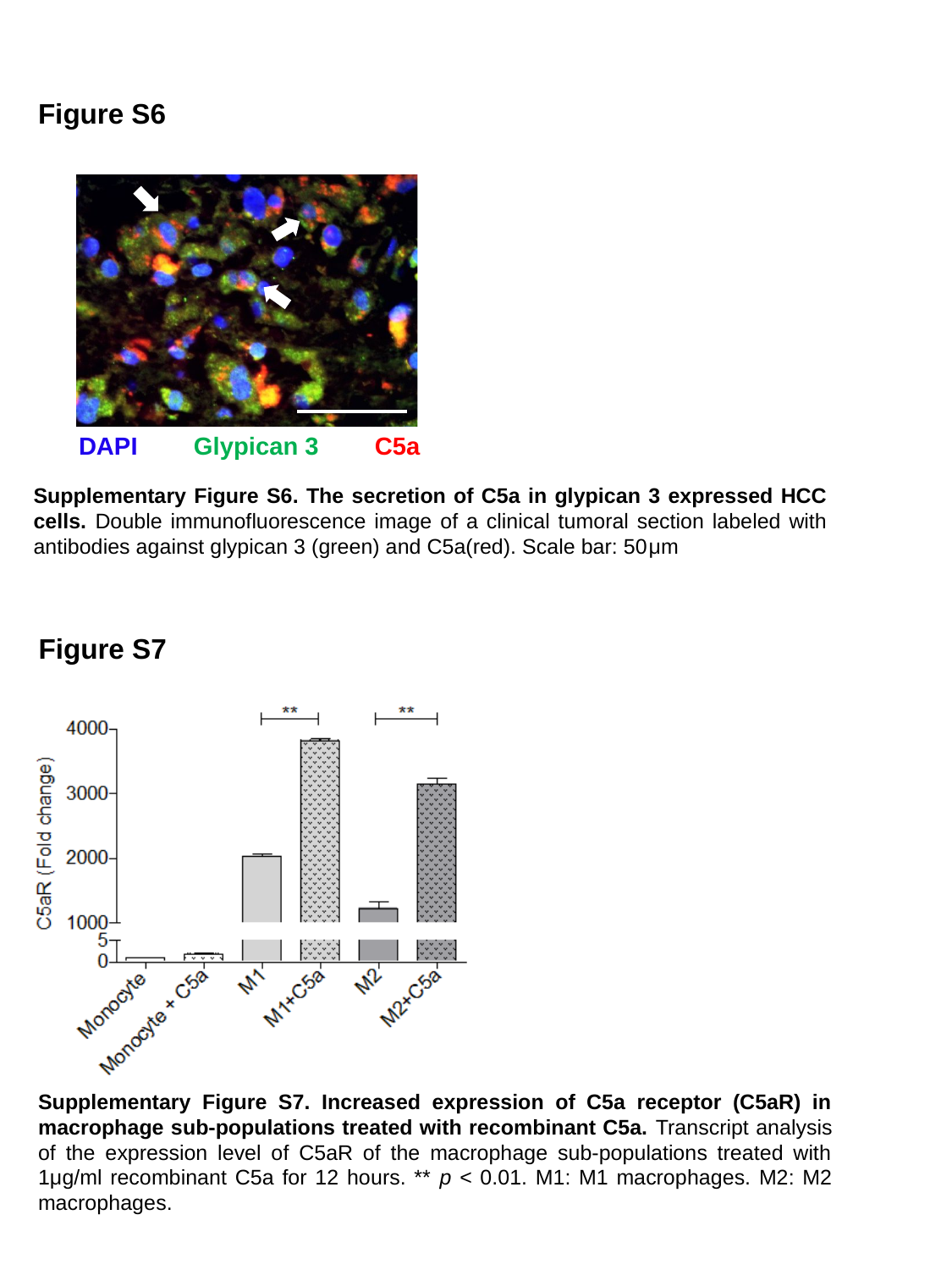

Figure S6
DAPI Glypican 3 C5a
Supplementary Figure S6. The secretion of C5a in glypican 3 expressed HCC cells. Double immunofluorescence image of a clinical tumoral section labeled with antibodies against glypican 3 (green) and C5a(red). Scale bar: 50μm
Figure S7
Supplementary Figure S7. Increased expression of C5a receptor (C5aR) in macrophage sub-populations treated with recombinant C5a. Transcript analysis of the expression level of C5aR of the macrophage sub-populations treated with 1μg/ml recombinant C5a for 12 hours. ** p < 0.01. M1: M1 macrophages. M2: M2 macrophages.

## Slide 6
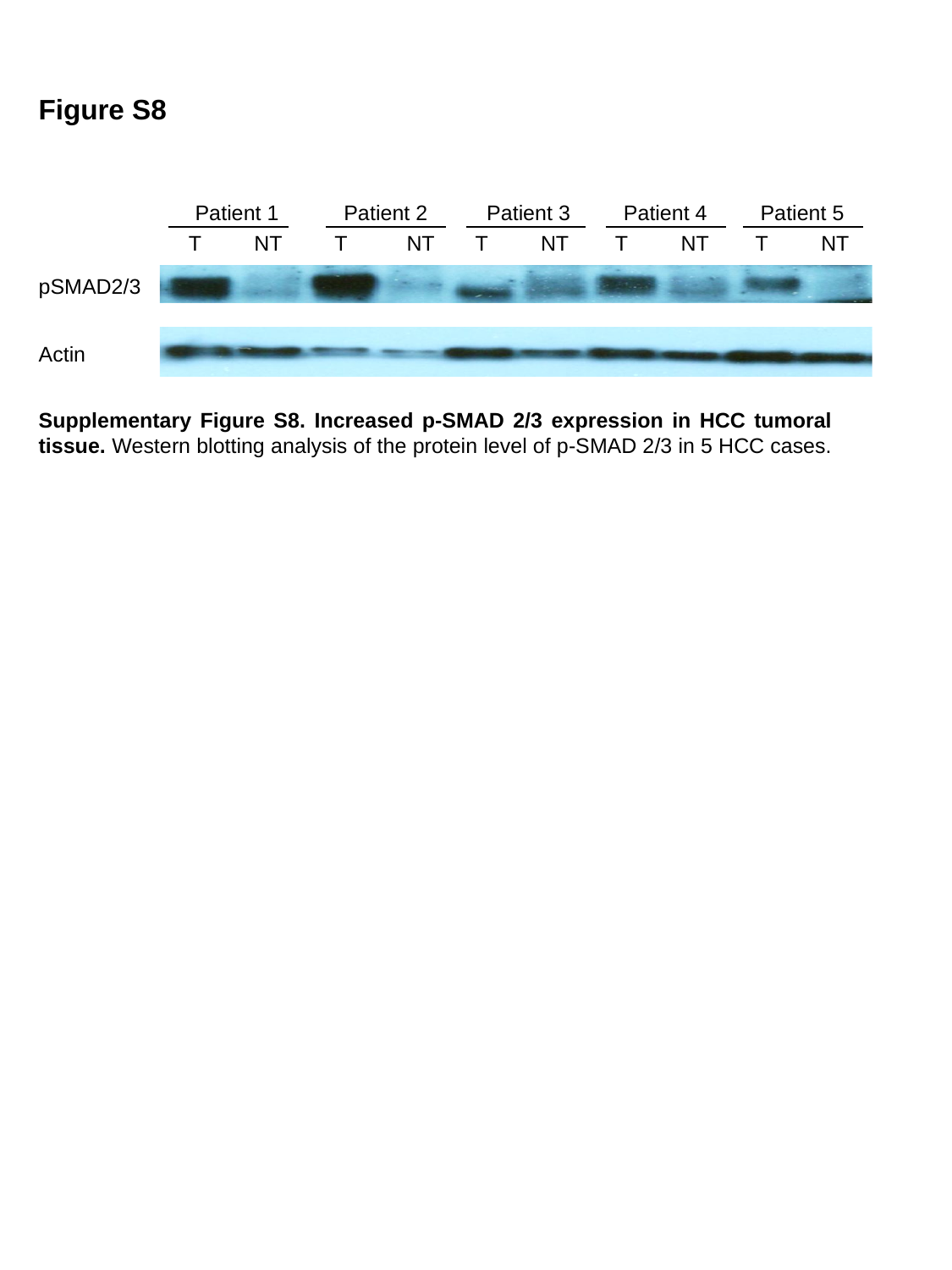

Figure S8
Patient 1 Patient 2 Patient 3 Patient 4 Patient 5
T NT T NT T NT T NT T NT
pSMAD2/3
Actin
Supplementary Figure S8. Increased p-SMAD 2/3 expression in HCC tumoral tissue. Western blotting analysis of the protein level of p-SMAD 2/3 in 5 HCC cases.
