# Supplementary material for: Type III TGF-β Receptor Down-Regulation Promoted Tumor Progression via Complement Component C5a Induction in Hepatocellular Carcinoma
Source: Cancers (Basel). 2021 Mar 25;13(7):1503. doi: 10.3390/cancers13071503 (PMC8037431; doi:10.3390/cancers13071503)
Supplement: Supplementary file 1 [file cancers-13-01503-s001.zip › Supplementary Tables.docx]

**Supplementary Tables**

**Table S1. Clinical characteristics of the studied population**

| **Number of cases 100** | |
| --- | --- |
| Age | 59^#^ (18-83) |
| Male | 77% |
| Hepatitis B positive | 73% |
| Smoking | 48% |
| Heavy alcoholic consumption | 8% |
| Bilirubin level (>20μmol/l) | 19% |
| Aspartate transaminase (>40U/L) | 64% |
| Alanine transaminase (>45U/L) | 58% |
| Alpha fetoprotein (>20ng/ml) | 53% |
| Child's grade | A: 91% ; B: 8% ; C: 1% |
| Liver cirrhosis | Normal: 39% ; Mildly: 62% ; Severely: 15% |
| HCC stage | I: 4% ; II: 24% ; III: 43% ; IV:29% |
| Tumor recurrence | 56% |
| Size of tumor (mm) | 6.65^#^ (0.9-20.2) |
| Extra-hepatic metastasis | 23% |
| Disease free survival (months) | 19^#^ |

| #Median |
| --- |

**Table S2. Transcript analysis of C5a treated M1 and M2 macrophages**

| **M1 macrophages (+C5a)** | \| Fold Change \| \| --- \| \| (Compared to control) \| | **M2 macrophages (+C5a)** | \| Fold Change  (Compared to control) \| \| --- \| \|  \| |
| --- | --- | --- | --- | --- | --- | --- | --- |
| IL-4 | 7.4909  5.21  3.5626  3.5179 | FASLG | 63.0185 |
| BMP-7 | 5.21 | TNFRSF11B | 28.5949 |
| IL-3 | 3.5626 | IL11 | 12.6013 |
| CCL-21 | 3.5179 | LTA | 9.9088 |
| ADIPOQ | 1.614 | XCL1 | 8.9518 |
| IL-22 | 1.5899 | IL22 | 5.5872 |
| TNFSF-11 | 1.5467 | IL17F | 4.8118 |
| IL-2 | 1.3923 | IL13 | 4.3993 |
| IL-10 | 1.3709 | CSF2 | 4.3724 |
| IL-27 | 1.3475 | IL21 | 3.4895 |
| CCL-17 | 1.3415 | IL3 | 3.4195 |
| THPO  MSTN | 1.3229 | CCL8 | 3.1351 |
| MSTN | 1.2774 | IL12B | 2.9232 |
| IL-17F | 1.2438 | THPO | 2.8002 |
| IL-17A | 1.243 | BMP7 | 2.7733 |
| IFNA-2 | 1.1996 | IL9 | 2.4232 |
| CCL-7  IL21 | 1.1653 | IL27 | 2.2907 |
| IL-21 | 1.1622 | LIF | 2.2071 |
| CCL-2 | 1.1268 | CXCL1 | 2.1877 |
| CNTF | 1.1234 | CXCL2 | 2.1332 |
| CXCL-11 | 1.0896 | BMP2 | 2.1066 |
| CSF-1 | 1.0815 | IFNG | 2.0731 |
| OSM | 1.0537 | PPBP | 2.068 |
| IL-15  IL7 | 1.033 | OSM | 2.0293 |
| IL-7 | 0.0319 | CCL1 | 1.9874 |
| CCL-1 | 1.0294 | CCL2 | 1.9328 |
| TGFB-2 | 1.0228 | LTB | 1.8797 |
| CCL-13 | 1.0142 | CXCL5 | 1.8433 |
| IL-1RN | 1.0113 | IL17A | 1.7258 |
| CCL-8 | 1.003 | TNF | 1.7186 |
| IL-11 | -1.0126 | CCL21 | 1.6725 |
| TNFSF-10 | -1.0128 | IL16 | 1.6181 |
| CSF-3 | -1.0163 | CCL3 | 1.6063 |
| C5 | -1.0202 | CCL20 | 1.6053 |
| IL-16 | -1.0202 | TNFSF11 | 1.5531 |
| TNF | -1.0461 | CX3CL1 | 1.5269 |
| MIF | -1.0625 | CXCL9 | 1.4999 |
| CD-40LG | -1.0702 | NODAL | 1.4951 |
| CCL-3 | -1.0729 | IL12A | 1.4626 |
| IL-1A | -1.0834 | CXCL16 | 1.4499 |
| IL-6 | -1.0973 | IL6 | 1.4379 |
| CXCL-10 | -1.1027 | MSTN | 1.4341 |
| CCL-18 | -1.1066 | GPI | 1.4076 |
| CX-3CL-1 | -1.1116 | CXCL8 | 1.3998 |
| BMP-4 | -1.1127 | IL1A | 1.3209 |
| TNFSF-13B | -1.13333 | IL1B | 1.149 |
| GPI | -1.139 | CCL7 | 1.177 |
| IL-18 | -1.1416 | TNFSF10 | 1.1606 |
| CXCL-9 | -1.1553 | CSF1 | 1.142 |
| CSF-2 | -1.1589 | BMP4 | 1.1212 |
| CCL-5 | -1.1643 | CXCL12 | 1.1009 |
| IL-23 | -1.2076 | SPP1 | 1.074 |
| PPBP | -1.2182 | CD40LG | 1.0645 |
| CXCL-8 | -1.2204 | CCL13 | 1.0077 |
| CXCL-12 | -1.2433 | CCL19 | 1.0077 |
| CXCL-1 | -1.2619 | IL4 | 1.0077 |
| SPP-1 | -1.2782 | MIF | 1.0072 |
| CCL-24 | -1.2827 | TNFSF13B | 1.0036 |
| CXCL-16 | -1.2982 | CSF3 | -1.013 |
| IFNG | -1.3097 | IL15 | -1.0202 |
| IL-5 | -1.3144 | IL18 | -1.0296 |
| CXCL-2 | -1.3507 | CNTF | -1.0332 |
| LTB | -1.3715 | CXCL10 | -1.0413 |
| IL-24 | -1.4049 | VEGFA | -1.0635 |
| CXCL-5 | -1.4217 | IL24 | -1.0848 |
| IL-9 | -1.4352 | CXCL11 | -1.116 |
| CXCL-13 | -1.4924 | TGFB2 | -1.1201 |
| NODAL | -1.4999 | IL1RN | -1.1676 |
| VEGFA | -1.533 | C5 | -1.1697 |
| BMP-2 | -1.6327 | CCL5 | -1.1783 |
| CCL-20 | -1.6381 | IL10 | -1.2001 |
| CCL-22 | -1.6433 | CCL24 | -1.236 |
| IL-12B | -1.6786 | CCL22 | -1.2596 |
| BMP-6 | -1.7063 | BMP6 | -1.2653 |
| IL-1B | -1.7071 | CCL18 | -1.3765 |
| LTA | -1.7627 | IL23A | -1.389 |
| CCL-19 | -1.8435 | CCL11 | -1.4727 |
| CCL-11 | -1.9125 | CCL17 | -2.7681 |
| LIF | -1.9722 | ADIPOQ | -3.1386 |
| IL-13 | -2.9618 | CXCL13 | -3.448 |
| IL-12A | -2.9718 | IL5 | -4.0752 |
| TNFRSF-11B | -3.3602 | IFNA2 | -4.2734 |
| FASLG | -11.3006 | IL7 | -4.5705 |
| XCL-1 | -55.5425 | IL2 | -14.6347 |

Fold change of cytokine/chemokine determined by Qiagen PHAS150Z RT^2^ Profiler^TM^ Human PCR array. Fold change > ±2.0 considered to be significant.
